# First Case of Acute Poisoning with Amiodarone and Flecainide in Attempted Suicide Successfully Managed with Lipid Emulsion Therapy in the Emergency Department: Case Report and Literature Review

**DOI:** 10.3390/healthcare9060671

**Published:** 2021-06-04

**Authors:** Cristina Bologa, Catalina Lionte, Alexandra Popescu, Victorita Sorodoc, Laurentiu Sorodoc

**Affiliations:** 1 Internal Medicine and Clinical Toxicology Department, “Grigore T. Popa” University of Medicine and Pharmacy, 700115 Iasi, Romania; cristina.bologa@umfiasi.ro (C.B.); victorita.sorodoc@umfiasi.ro (V.S.); laurentiu.sorodoc@umfiasi.ro (L.S.); 2 2nd Medical Clinic, “Sf. Spiridon” Emergency Clinical County Hospital, 700111 Iasi, Romania; alexandraa.lita@yahoo.com

**Keywords:** suicide, poisoning, amiodarone, flecainide, cardiotoxicity, lipid emulsion therapy, Emergency Department

## Abstract

Acute antiarrhythmics poisoning represents a challenge in the Emergency Department (ED). These patients often develop malignant arrhythmias in need of exceptional therapeutic measures in the ICU. We report a 47-year-old patient admitted to the ED 5 h after the ingestion of a large dose of amiodarone and flecainide in a suicide attempt. During their ED stay, the patient developed signs of cardiotoxicity evidenced by electrocardiogram and ventricular arrhythmias. The toxicological results showed a level of 4.8 mg/L amiodarone and 2.98 mg/L flecainide. He was successfully treated in the ED using a large dose of sodium bicarbonate and lipid emulsion therapy. After hospital admission, he remained stable, with no need for exceptional therapeutic measures such as mechanical circulatory support, cardiac pacing or ECMO. We emphasize the importance of an early start of pharmacological therapies in the ED, which might improve the outcome in antiarrhythmic acute poisoning.

## 1. Introduction

Antiarrhythmic drugs are widely used in clinical practice. Antiarrhythmic drug therapy carries an understood risk for toxic side effects, even with therapeutic doses. Flecainide induces ventricular tachycardia (VT), which is often resistant to direct current cardioversion because the intense sodium channel blockade increases the voltage threshold for cellular depolarization [1]. Amiodarone prolongs the QT interval, causes conduction disturbances and VT. Amiodarone is also related to exacerbations of VT and an increased defibrillation threshold [2,3]. A combination of these two antiarrhythmics in intentional overdose could lead to dramatic cardiotoxicity. 

A review of the literature showed no report of a case with a poisoning including an association of amiodarone and flecainide. We found only two case reports of amiodarone intentional overdose: the first case was of a patient who ingested 8000 mg amiodarone in a suicide attempt, where the blood concentration was 1.1 mg/L, managed with supportive measures; the second case was of a patient with mixed overdose of amiodarone, diltiazem and metoprolol, with a serum level of amiodarone of 2.7 mg/L, in need of intensive care unit (ICU) therapy [4,5]. In flecainide overdose, there is a need for exceptional therapeutic interventions both in the Emergency Department (ED) and ICU [6,7,8,9].

We report the case of a patient who attempted suicide by ingesting a large dose of amiodarone and flecainide, with an electrocardiogram (ECG) depicting signs of cardiotoxicity and ventricular arrhythmias, which was managed successfully in the ED, after hypertonic sodium bicarbonate administration and initiation of lipid emulsion therapy (LET).

After ED admission of a patient with mixed antiarrhythmic drugs poisoning, ECG monitoring, recognition of signs of cardiotoxicity and early initiation of hypertonic sodium bicarbonate and LET may improve the outcome and avoid exceptional therapeutic measures in the ICU. 

## 2. Case Presentation

A 47-year-old patient was admitted to the ED for fatigue, anxiety, headache. The patient declared the ingestion of 2000 mg amiodarone and 5000 mg flecainide in a suicide attempt, 5 h prior to ED arrival. Glasgow Coma Scale score was 12, BP 110/80 mmHg, heart rate 71 bpm.

**Figure 1:**
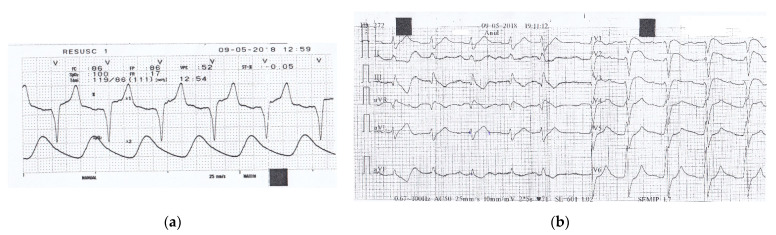
(**a**) Monitor recording during transportation to the first hospital shows **a** sinus rhythm of 86/min, a prolonged PR interval of 240 msec with normal QRS complex of 110 msec.; (**b**) 12-lead ECG recorded in the local hospital, 3 h after the ingestion of drugs, shows irregular sinus rhythm of 71/min, with a prolonged PR interval of 280 msec, a wide QRS complex of 160 msec, and slightly prolonged QTc interval of 479 msec.

The patient had an episode of atrial fibrillation converted to sinus rhythm with flecainide two years before, had been subjected to a radiofrequency catheter ablation with the isolation of the pulmonary veins one year earlier for atrial flutter, with successful restoration and maintenance of sinus rhythm, and took amiodarone 100 mg daily since. He had a history of depression, but he had quit the specific therapy for several months. During transportation, the monitor recorded a sinus rhythm of 86/min, a prolonged PR interval 240 msec with a normal QRS complex (Figure 1). He was first admitted to a local hospital, where he received an initial dose of activated charcoal and normal saline solution, then referred to our ED for specific therapy. The ECG recorded upon first medical contact showed an irregular rhythm, a first-degree atrioventricular (AV) block with a wide QRS complex.

Upon admission to our ED, arterial blood gases (ABG), liver and kidney panel, alkaline phosphatase and LDH were normal, WBC 12.000/mmc, blood glucose 170 mg/dL, and urine toxicological screen negative. Cardiac ultrasound excluded a structural disease. Cardiac biomarkers were within normal range.

Given the large dose of antiarrhythmics ingested, we began sodium bicarbonate 8.4% administration, up to 500 mEq/L (Table 1). A second ABG showed pH 7.53 and a sodium concentration >150 mEq/L. We initially administered 1 g/kg activated charcoal, followed by 25 g every 4 h for 12 h, to reduce the gastrointestinal absorption of flecainide and increase the elimination of amiodarone, which enters in an enteral-hepatic circuit [10,11].

**Figure 2:**
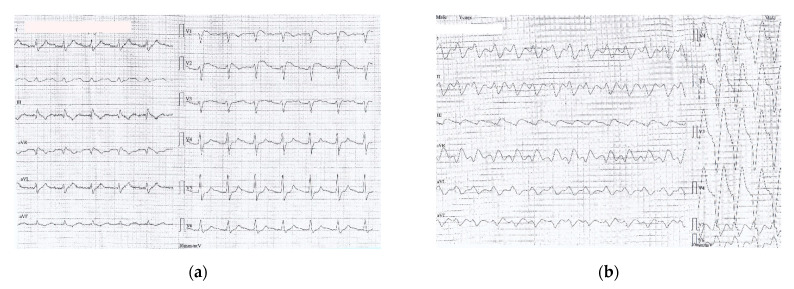

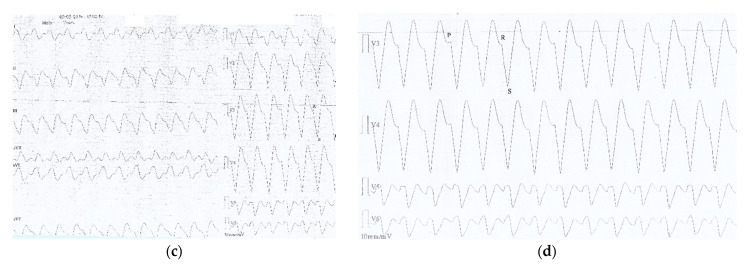
(**a**) ECG recorded one hour after ED admission (6 h after drugs ingestion): sinus rhythm 67/min, prolonged PR interval 360 msec, wide QRS complex 200 msec, prolonged QTc 549 msec, negative T wave DIII, aVF, with a coved ST segment elevation in V1-V2; (**b**) ECG recorded two hours after ED admission (7 h after drug ingestion) showing a slightly irregular rhythm, no visible P waves, sinusoidal pattern, wide LBBB pattern-QRS complexes at a rate of 97/min (**c**) ECG recorded two and a half hours after ED admission (7 and a half hours after drug ingestion): VT 110/min (wide QRS complex tachycardia, AV dissociation, negative concordance in precordial leads, R to nadir S 160 msec); (**d**) ECG detail (magnified precordial leads) showing VT criteria: AV dissociation and R to nadir S > 100 msec.

One hour after admission, the ECG changed significantly and VT occurred later (Figure 2). First, we administered MgSO4 2 g over 20 min, then a bolus of 1.5 mL/kg lipid emulsion (Intralipid^®^ 20% IV fat emulsion) was pushed over 2–3 min, which was repeated, followed by 0.25 mL/kg/min infusion over one hour. 

**Figure 3:**
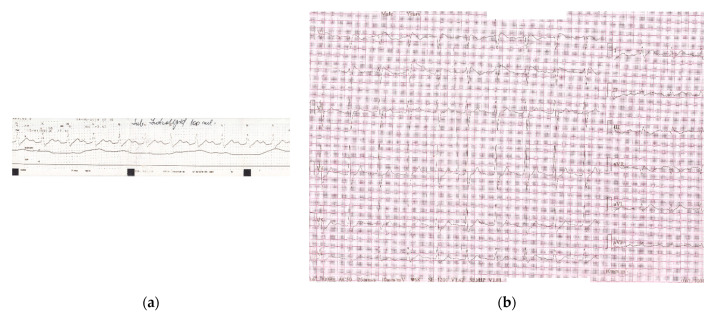
(**a**) Monitor recording during first 100 mL Intralipid^®^ solution revealing reversal of ECG changes: sinus rhythm 68/min with distinct visible P waves, prolonged PR interval 320 msec, wide QRS complex 160 msec, QT interval 480 msec; (b)12-lead ECG recorded after administration of the first 100 mL Intralipid^®^: sinus rhythm 71/min, first degree AV block (PR interval 320 msec), wide QRS complex RBBB-type 180 msec, disappearance of negative T waves in aVF, corrected QTc interval 479 msec.

ECG changes showed significant improvement in 20 min after only 100 mL Intralipid^®^ (Figure 3). 

**Figure 4:**
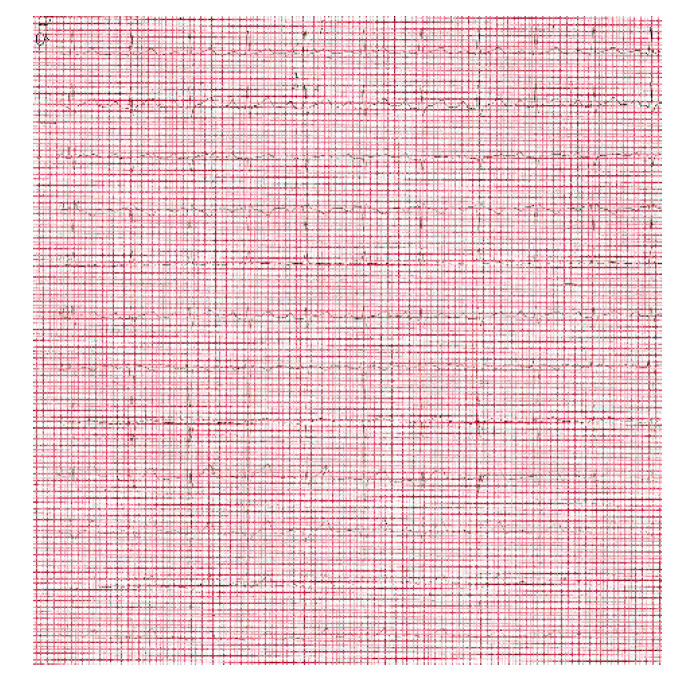
ECG upon discharge, shows sinus rhythm 90/min, with normal PR, QTc intervals and QRS complex.

During his ED stay, the patient remained hemodynamically stable. He was admitted to a medical ward, had no other complications, and was released home 48 h later. The ECG returned completely to normal baseline (Figure 4) and the psychiatric medication was properly re-initiated. 

The toxicological results, which were not available during ED stay, showed in our patient a level of 4.8 mg/L amiodarone and 2.98 mg/L flecainide from the blood sample obtained upon ED admission (5 h after drug ingestion). These levels were extremely high compared with the usual therapeutic range, which is 1–2.5 mg/L for amiodarone and 0.2–1 mg/L for flecainide [12].

## 3. Discussion

**Figure 5:**
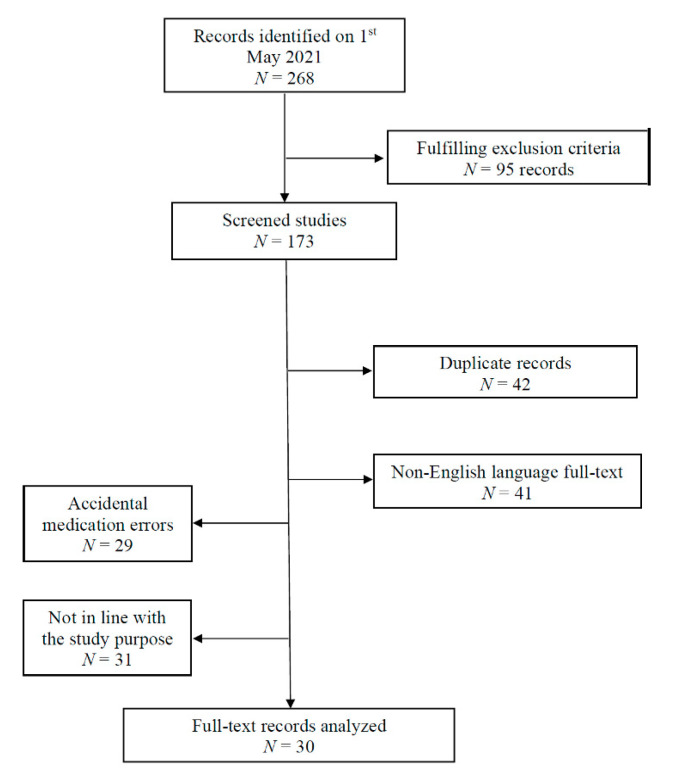
Flowchart illustrating studies included and excluded in this literature review.

An extensive search of the literature was performed with the journal search engines Thompson ISI—Web of Science, EMBASE, EBSCO, Scopus and PubMed. We used the MeSH terms suicide, amiodarone, flecainide, AND overdose, intoxication, or poisoning in different permutations. Additionally, we examined the citations of all resulting articles for any additional relevant references. Each article was reviewed, and case reports, which included and pictured a 12-lead ECG performed during intoxication, as well as references to intentionality of the poisoning, time to hospital admission, dosage and/or serum level, therapy administered and setting, were included for analysis. A summary of the details of the included papers is reported in Table 2. The exclusion criteria were: animal and in-vitro studies, forensic and analytical studies, original articles that were not in the English language, as well as comments, editorials, posters, abstracts and letters to the Editor. Those articles reporting flecainide or amiodarone accidental medication errors, and reports with incomplete data were also excluded (Figure 5).

For each article, we analyzed relevant demographic and clinical data from the case. Next, a thorough analysis of each ECG pattern was performed. We also analyzed therapeutic approaches and the department where the treatment was conducted.

To our knowledge, this is the first report of a suicide attempt after intentional ingestion of large doses of amiodarone and flecainide with severe cardiotoxicity and a favorable evolution after LET in the ED. There are reports of accidental overdose in patients treated chronically with flecainide, especially in elders with hepatic or renal comorbidities, that were resolved in the ICU [32,34,37]. There are also reports of flecainide-induced therapy resistant ventricular fibrillation followed by cardiac arrest, successfully treated with cardiopulmonary resuscitation and advanced life support, where amiodarone was used as part of the protocol [19,29,32]. From the studies analyzed, only in one case of accidental flecainide overdose was the patient managed successfully in the ED with Na bicarbonate [36]. All the other reports involved patients in need of exceptional measures, started in the ED and continued in ICU/CCU (Table 2). 

In our review, we found two reports of deceased patients, both after ingestion of 10–12 g flecainide. The patients had an unfavorable outcome despite the administration of sodium bicarbonate (250 mL) and ACLS therapies, including ECMO (Table 2) [17,24]. Another patient, with a moderate dose of flecainide ingested in association with other drugs, also had an unfavorable evolution after ED and CCU therapy, which included CPR, administration of MgSO4, electrolyte correction, LET and defibrillation [35]. 

Flecainide toxicity is rare but potentially fatal; at higher doses, flecainide toxicity can result in hemodynamic collapse, with a mortality rate of up to 22.5% [38]. Amiodarone inhibits several cytochrome P450 pathways, thus increasing serum concentrations of drugs such as statins, calcium channel blocking agents, tacrolimus, quinidine, fentanyl and flecainide. Plasma concentrations >2.5 mg/L have been associated with increased risk of toxicity [39].

Antiarrhythmic drugs’ toxicity can be classified based either on the clinical features, or on ECG changes (Table 3). 

Flecainide overdose determines nonspecific symptoms (nausea, vomiting, headache), seizures bradycardia, QRS widening and ventricular arrhythmias [10,11,30,40]. Flecainide is a lipophilic drug that acts by strongly blocking the sodium channels, delaying their reactivation without impeding repolarization. Flecainide binds and opens sodium channels in a dose-dependent manner. In patients without structural heart disease, flecainide slows the conduction and favors reentry, creating areas of functional block, thus producing reentry arrhythmias. Among the class Ic antiarrhythmics, flecainide is the most difficult to detach from the sodium channels, delaying their reactivation [1,11]. The plasmatic peak is 3–4 h after flecainide ingestion. After absorption, 70% of the dose ingested is metabolized in the liver, while 30% is eliminated unchanged by the kidney. In patients with renal impairment, the total clearance of this drug might fall by approximately 40% [41]. The elimination time is dose-dependent, and is increased by urine alkalinization [5,7].

Amiodarone is a class III antiarrhythmic with all the properties of the four Vaughn-Williams antiarrhythmic classes. After oral administration, the maximal plasmatic concentration is reached 3–7 h later [11,40]. Amiodarone affects bioavailability, binding with plasmatic proteins, metabolism by liver cytochromes and kidney elimination of co-administered antiarrhythmics. Thus, amiodarone significantly increases the plasma level of flecainide, leading to severe cardiotoxicity. Amiodarone is likely to cause polymorphic ventricular dysrhythmias and the effect is potentiated by hypokalemia or hyperglycemia [42,43,44].

Large doses of sodium bicarbonate should be administered in flecainide overdose to counteract cardiotoxicity by plasma alkalinization, which decreases the free concentration of the drug, promotes drug dissociation from the sodium channels and increases extracellular concentration of sodium ions that displace the drug from the receptor sites [1,30,45]. The dose of the hypertonic sodium bicarbonate is 1 mEq/kg bolus (range, 0.55–3.0 mEq/kg) followed by infusion of 15 to 20 mEq/h, maintaining a target pH of 7.50 to 7.60 [1].

LET is an adjunctive measure, which will sequestrate both flecainide and amiodarone, decreasing the drug available to block sodium channels [30,45] The mechanism of action of LET is unclear; however, it is postulated that it follows the mechanisms of the “lipid-sink theory” whereby LET acts to sequester lipophilic drugs, such as flecainide, thereby reducing toxic activity on cardiac myocytes [46]. The administration of LET compartmentalizes the offending drug into a lipid phase and away from the target receptors. The drugs with a high lipid solubility favor the lipid partition and leave the serum, thus lower serum concentrations facilitate the removal of the offending agent from tissues by the generation of a concentration gradient [47,48]. The second mechanism suggests that LET exerts a positive inotropic effect with more efficient metabolization of fatty acids [25]. 

To our knowledge, ten cases have been published in the literature regarding the use of LET as part of a complex therapeutic protocol initiated in the ED, continued in ICU/CCU, for severe cardiotoxicity in flecainide overdose. LET was also used in one case of mixed poisoning including amiodarone, betablockers and calcium-channel blockers. In reviewed cases, LET was associated (in addition to treatment with high doses of sodium bicarbonate) with ACLS measures, CPR, pacing, CVVH and ECMO [6,7,26,28,31,34,35]. In a single case, in which the patient arrived at the ED 90 min after self-poisoning with a lower flecainide dose as compared with our patient, with a serum level of flecainide slightly over the therapeutic range, LET was involved as part of a pharmacological protocol that included atropine, MgSO4, Na bicarbonate for bradycardia, AV block and QTc prolongation. However, this case needed ICU surveillance [25].

Particular to our case was that it was recorded when, in our country, flecainide was not authorized by the National Agency for Medicines and Medical Devices. The use of LET immediately after occurrence of ventricular arrhythmias led to a significant improvement in QRS and QTc duration and the restoration of sinus rhythm, within 20 min, in the ED. 

While the evidence base for LET use in acute drug intoxication is evolving, the present evidence and recommendation supports the use of LET in lipophilic cardiotoxin intoxication when there is an immediate threat to life, and other therapies have proven ineffective [49,50]. 

Patients who do not respond to drug therapy could benefit from cardiac pacing, ECMO, or mechanical circulatory support [30,51]. ECMO is a temporizing measure to allow for cardiac recovery and drug elimination and should be reserved for refractory hemodynamic compromise [52].

Extra-corporeal life support (ECLS) provides respiratory support and also crucially maintains cardiac output, preventing end-organ damage and restoring vital organ perfusion, thereby enabling renal drug elimination, hepatic drug metabolism and drug redistribution. The length of the ECLS support may be determined by serum flecainide level and cardiac stability [26].

The first step in the management of a mixed antiarrhythmic overdose should be recording an ECG to identify QRS widening, QTc prolongation, or atrio-ventricular blocks. The second step should be ABG determination and decontamination measures. Then, pharmacological therapies that proved to be beneficial, such as hypertonic sodium bicarbonate or LET, should be initiated early in the ED, when the first signs of cardiotoxicity occur. 

We reported the first case of intentional amiodarone and flecainide poisoning, drugs that interact, both leading to life-threatening cardiotoxicity. The patient was admitted to the ED 5 h after drug ingestion and initially had a nonspecific clinical picture. ECG signs of cardiotoxicity occurred 6 h after ingestion, although immediate measures for decontamination and hypertonic sodium bicarbonate were initiated. Alkalinization with a pH over 7.5 and increased extracellular sodium contribute to flecainide dislocation from cardiomyocytes and decrease serum free flecainide level [45]. LET was given after failure of other therapies, prior to cardiovascular collapse, as the VT occurred. ECG changes in our patient were improved within 20 min of LET. The patient remained hemodynamically stable with an uneventful evolution during the next 48 h of hospitalization. 

We undertook the present study with the goal of reviewing the reports of amiodarone and flecainide acute poisoning in humans, with evidence of ED therapies used, their effect, and failure as a treatment for poisoning. We aimed to distinguish which variables might explain failures and successes in either time of administration, substances, or toxic load.

## 4. Conclusions

With this case and the data reported in the literature, the authors want to point out that: (a) emergency physicians need to proceed to close cardiac monitoring of the patients with mixed antiarrhythmics poisoning; (b) administration of life-saving therapies, such as hypertonic sodium bicarbonate and LET after first cardiotoxicity signs, is feasible in the ED and should help to avoid the need for exceptional measures in the ICU.

Since antiarrhythmics are widely used in clinical practice, further research on pharmacological therapies and antidotes is crucial for taking an important preventive action. 

## Figures and Tables

**Table 1:** Patient’s evolution during ED admission and therapy provided.

Time after Exposure	Investigations and Therapy Administered	Patient Evolution
5 h after drug ingestion (ED admission)	Monitoring, blood samples drawn for hematological, biochemical, toxicological tests ECG recording IV line with saline solution 500 mL, sodium bicarbonate 8.4% 50 mL	Symptoms: fatigue, headache Signs: GCS 12 BP 110/80 mmHg HR 71 bpm SaO2 95% room air
6 h after drug ingestion (First hour in ED)	Activated charcoal 50 g orally Cardiac ultrasound Second ECG recording showing significant changes (Figure 2a) Additional sodium bicarbonate 8.4% 250 mL	GCS 13 BP 103/67 mmHg HR 67 bpm SaO2 97% (oxygen 2l nasal canula) Symptoms: dizziness, palpitation
7–8 h after drug ingestion (Second to third hour in ED)	Sodium bicarbonate 8.4% 200 mL ECG recording showing wide QRS complex rhythm (Figure 2b) IV administration of MgSO4 2 g over 20 min ECG recording showing VT (Figure 2c,d) A bolus of 1.5 mL/kg lipid emulsion (Intralipid^®^) pushed over 2–3 min, which was repeated, followed by 0.25 mL/kg/min infusion over the next hour ECG changes improved after first 20 min of LET (Figure 3a,b)	GCS 13 BP 95/65 mmHg HR variable 97–110 bpm SaO2 97% (oxygen 2l nasal canula) Symptoms: fatigue, palpitations
9 h after drug ingestion (Four hours after ED admission)	ECG changes restored to admission pattern LET completed Second dose of 25 g activated charcoal Admission for further monitoring and therapy in a medical ward	GCS 14 BP 108/78 mmHg SaO2 96% room air Symptoms: fatigue, anxiety

GCS, Glasgow Coma Scale score; VT, ventricular tachycardia: LET, lipid emulsion therapy.

**Table 2:** Characteristics and main findings of eligible studies.

Author(s), Year	Demographic/ Time/ Intent	Medication/Dose/ Serum Level * /Co-ingestions	ECG Patterns	Therapy/ Setting	Outcome
Bonati et al., 1983 [4]	W, 20-y/ 12 h/ Intentional	A/8000 mg/ 1.1 mg/L /no	Bradycardia, prolonged QTc	Supportive/ Medical department	Survival
Winkelmann et al., 1987 [13]	W, 28-y/ 2 h/ Intentional	F/3800 mg/ 3.7 mg/L /diazepam, loperamide, ethanol	Monomorphic VT, polymorphic VT	ACLS, Na bicarbonate, Na chloride, physostigmine salicylate, GL, AC/ ICU	Survival
Yasui et al., 1997 [8]	W, 20-y/ 90 min/ Intentional	F/NR/ 5.45 mg/L /ethanol	Idioventricular rhythm 40/min	Hypertonic saline, pacing, CBS/ ED, ICU	Death
Goldman et al., 1997 [14]	M, 16-y/ 30 min/ Intentional	F/4000 mg/ NR /no	VT, irregular WCT, prolonged QTc	Cardioversion, Na bicarbonate, lidocaine, GL, AC/ ED, ICU	Survival
Hanley et al., 1998 [15]	W,15-y/ 1 h/ Intentional	F/9000 mg/ 5 mg/L /no	Wide QRS irregular bradycardia, VT, prolonged QTc, AV block	Atropine, GL, AC, adrenaline, ECV, lidocaine, isoprenaline, pacing/ ICU	Survival
Lovecchio et al., 1998 [16]	1. W, 38-y/ 40 min/ Intentional 2. M, 61-y/ 8 h/ Intentional	1. F/1000 mg/ 2.18 mg/L /caffeine 2. F/3500 mg/ 3 mg/L /digoxin, losartan, ranitidine	1. Irregular rhythm, wide QRS 2. AF 105/min, wide QRS	1. GL, AC, supportive, Na bicarbonate/ED, ICU 2. repeated boluses of Na bicarbonate/ NR	1.Survival 2.Survival
Brazil et al., 1998 [17]	M, 36-y/ 6 h/ Intentional	F/10,000 mg/ 3.32 mg/L /no	Wide QRS, VT, PEA cardiac arrest	Na bicarbonate, ACLS/ ED, CCU	Death
Corkeron et al., 1999 [18]	W,20-y/ 15 min/ Intentional	F/4000 mg/ 4.25 mg/L /paracetamol	Irregular wide complex rhythm, PEA	CPR, Na bicarbonate, AC, adrenaline, pacing, CBP, CVVH/ ED, ICU	Survival
Auzinger et al., 2001 [9]	M, 30-y/ 1 h/ Intentional	F/6000 mg/ 20.52 mg/L /NR	Wide complex bradycardia	CPR, Na bicarbonate, pacing, ECMO/ ED, ICU	Survival
Siegers & Board, 2002 [19]	W,45-y/ NR/ Intentional	F/2000 mg/ 0.85 mg/L /ethanol	Bradycardia, pulseless VT, VF	CPR, ACLS, GL, AC, Na bicarbonate, amiodarone/ ED, ICU	Survival
Hudson et al., 2004 [20]	M, 70-y/ NR/ Accidental	F/1500 mg/ 2.96 mg/L /no	Wide complex rhythm, Brugada-type STE	Repeated Na bicarbonate boluses, supportive/ Medical unit	Survival
Timperley et al., 2005 [21]	W,47-y/ NR/ Accidental	F/NR/ 2.34 mg/L /amitriptyline, losartan, amlodipine	AF, wide QRS, sine wave appearance	Alteplase (for presumptive ACS), dobutamine, adrenaline, IABP/ ED, CTU	Survival
Devin et al., 2007 [22]	W, 34-y/ 90 min/ Intentional	F/4500 mg/ 3.6 mg/L /no	Irregular wide complex rhythm	Supportive, sodium bicarbonate/ ED, ICU	Survival
Rognoni et al., 2009 [23]	W, 57-y/ NR/ Intentional	F/1800 mg/ 1.94 mg/L /no	RBBB, wide QRS, prolonged QTc	Supportive, AC, MgSO4, Na bicarbonate/ ED, ICU	Survival
Vivien et al., 2010 [24]	W,40-y/ 10 h/ Intentional	F/12,000 mg/ 34 mg/L /betaxolol	Wide QRS bradycardia, Brugada-type, asystole	CPR, Na bicarbonate, epinephrine, dobutamine, ECMO/ ED, ICU	Death
Stellpflug et al., 2011 [5]	W, 30-y/ 6 h/ Intentional	A/NR/ 2.7 mg/L /diltiazem, metoprolol	Paced rhythm, no change in interval/ segment length	Supportive, calcium, HDI, LET/ ED, ICU	Survival
Ellsworth et al., 2013 [25]	M, 51-y/ 90 min/ Intentional	F/2500 mg/ 1.8 mg/L /no	Bradycardia, 1st degree AV block, wide QRS, prolonged QTc	AC, Na bicarbonate, Atropine, MgSO4, LET/ ED, ICU	Survival
Sivalingam et al., 2013 [26]	W, 52-y/ NR/ Intentional	F/NR/ 4.13 mg/L /no	Profound bradycardia, wide QRS, PEA	Pacing, CPR, ACLS, AC, Na bicarbonate, LET, ECMO/ ED, ICU	Survival
Reynolds & Judge, 2015 [6]	W, 24-y/ NR/ Accidental	F/400 mg/ 11.085 mg/L /caffeine, levetiracetam	Wide QRS bradydysrhythmia, PEA cardiac arrest	Na bicarbonate, vasopressors, pacing, LET, ECMO/ ED, ICU	Survival
Mandawat et al. 2015 [7]	W, 33-y/ NR/ Intentional	F/1800 mg/ NR /no	Wide complex rhythm, VT, prolonged QTc, PEA	Na bicarbonate, LET, ACLS, ECMO/ ICU	Survival
Williamson et al., 2015 [27]	W, 18-y/ 45 min/ Intentional	F/1200 mg/ NR /no	Wide QRS bradycardia, WCT	Atropine, Na bicarbonate, dobutamine, epinephrine, MgSO4/ ED, medical ward	Survival
Mukhtar et al., 2015 [28]	W, 13-y/ 90 min/ NR	F/900 mg/ 2.699 mg/L /bisoprolol	1st degree AV block, RBBB, prolonged QTc, VT, TdP, Brugada-like syndrome, VF	Na bicarbonate, MgSO4, glucagon, CPR, LET, pacing/ ED, ICU	Survival
Jung et al., 2016 [29]	M, 20-y/ 1 h/ Intentional	F/5000 mg/ NR /no	Irregular wide QRS bradycardia, pulseless VT, prolonged QTc, TdP, VF	Supportive, dopamine, CPR, Na bicarbonate, MgSO4, lidocaine, GL, AC, amiodarone, ECV/ ED, ICU	Survival
Vu et al., 2016 [30]	M, 23-y/ NR/ Intentional	F/NR/ 2 mg/L /amphetamine	Wide complex rhythm, pulseless VT, VF	CPR, Na bicarbonate, pacing, ECMO/ ED, CCU	Survival
Mullins et al., 2017 [31]	1. M, 49-y/ NR/ Intentional 2. M, 69-y/ 70 min/ Intentional	1. F/2400 mg/ NR /no 2. F/1000 mg/ NR /clonazepam, ropinirole	1. Bradycardia, asystole, wide QRS 2. Wide complex irregular rhythm, prolonged QTc, wide QRS	1.Atropine, glucagon, CPR, dopamine, Na bicarbonate, LET/ ED 2. Na bicarbonate, ALS, LET/ ED, CCU	1.Survival 2.Survival
Apfelbaum et al. 2018 [32]	W, 86-y/ NR/ Accidental	F/NR/ 1.39 mg/L /no	Wide complex rhythm, pacemaker spikes	Supportive, Amiodarone, Na bicarbonate, reprograming pacemaker/ ED, ICU	Survival
Bodziock et al., 2018 [33]	M, 48-y/ NR/ Intentional	F/NR/ 3.03 mg/L /no	Irregular sinusoidal waveforms, prolonged QTc, wide QRS, ST-depressions, T-wave inversions	Pacing, epinephrine, supportive, metoprolol/ ED, Cardiology service	Survival
Heldens et al., 2019 [34]	W, 68-y/ NR/ Accidental	F/NR/ 2.44 mg/L /no	Extreme broad QRS complexes, loss of pacemaker capture	Na bicarbonate, CVVH, LET/ ED, CCU	Survival
Gaylor et al., 2019 [35]	W, 30-y/ NR/ Intentional	F/1500 mg/ 2.01 mg/L /ondansetron	Cardiac arrest, wide complex arrhythmia, prolonged QTc	CPR, Mg, K, epinephrine, defibrillation, LET/ ED, CCU	Death
Venkataraman 2020 [36]	M, 70-y/ 4 h/ Accidental	F/900 mg/ NR /no	WCT, RBBB, anterior ST segment elevation	Na bicarbonate/ ED	Survival

*, serum level reported for each case was transformed in mg/L; W, woman; A, amiodarone; F, flecainide; VT, ventricular tachycardia; ACLS, advanced cardiac life support measures; GL, gastric lavage; AC, activated charcoal; ICU, intensive care unit; ED, Emergency Department; CBS, peripheral cardiopulmonary bypass support; AV, atrioventricular; ECV, electrical cardioversion; M, male; AF, atrial fibrillation; NR, not reported; WCT, wide complex tachycardia; PEA, pulseless electrical activity; CCU, coronary care unit; CVVH, continuous veno-venous hemodiafiltration; CPR, cardiopulmonary resuscitation; ECMO, extracorporeal membrane oxygenation; VF, ventricular fibrillation; STE, ST segment elevation; ACS, acute coronary syndrome; IABP, intra-aortic balloon pump; CTU, cardio-thoracic unit; HDI, high-dose-insulin therapy; LET, lipid emulsion therapy; RBBB, right bundle branch block; TdP, torsade de pointes; ALS, advanced life support.

**Table 3:** Antiarrhythmic drugs toxicity (adapted from [10,11,12]).

Clinical Features		ECG Changes	
Hypotension	Amiodarone, dronedarone flecainide, ibutilide lidocaine, mexiletine, procainamide, propafenone, quinidine	Wide QRS	Amiodarone, dronedarone, disopyramide, flecainide, procainamide, propafenone quinidine
Heart failure	Disopyramide, flecainide, procainamide, propafenone, sotalol	Prolonged QTc	Amiodarone, dronedarone disopyramide, dofetilide, ibutilide, procainamide, sotalol, quinidine, flecainide, propafenone (only slight prolongation)
Seizures	Flecainide, lidocaine, mexiletine, procainamide, propafenone, quinidine	Ventricular arrythmias	Procainamide
Neurological symptoms and signs	Disopyramide, flecainide, lidocaine, mexiletine, procainamide, propafenone, quinidine	Prolonged PR	Procainamide
Anticholinergic symptoms and signs	Disopyramide, procainamide, quinidine	Torsade de pointes	Ibutilide, dofetilide, sotalol, quinidine, procainamide, disopyramide, amiodarone (rare)
Endocrine changes	Amiodarone (hypo/hyperthyroidism), quinidine, disopyramide (hypoglycemia)	Increased ventricular rate in atrial flutter	Quinidine, flecainide, propafenone
Pulmonary changes	Amiodarone (fibrosis, pneumonitis)		
Hematological/oncological changes	Amiodarone (hepatobiliary carcinoma), procainamide (hemorrhagic syndromes)		
Autoimmune manifestations	Procainamide (lupus-like syndrome, vasculitis)	Incessant ventricular tachycardia	Flecainide, propafenone, quinidine (rare)
Skin changes	Procainamide, propafenone		

## Data Availability

Not applicable.

## Abbreviations

ED	Emergency Department
ECMO	Extracorporeal Membrane Oxygenation
VT	ventricular tachycardia
ICU	intensive care unit
ECG	electrocardiogram
LET	lipid emulsion therapy
AV	atrioventricular
ABG	arterial blood gases
WBC	white blood cells
ACLS	advanced cardiac life support measures
GL	gastric lavage
AC	activated charcoal
CBS	peripheral cardiopulmonary bypass support
ECV	electrical cardioversion
AF	atrial fibrillation
WCT	wide complex tachycardia
PEA	pulseless electrical activity
CCU	coronary care unit
CVVH	continuous veno-venous hemodiafiltration
CPR	cardiopulmonary resuscitation
VF	ventricular fibrillation
STE	ST segment elevation
ACS	acute coronary syndrome
IABP	intra-aortic balloon pump
CTU	cardio-thoracic unit
HDI	high-dose-insulin therapy
RBBB	right bundle branch block
TdP	torsade de pointes
ALS	advanced life support
ECLS	extra-corporeal life support
